# Redundancy in microbiota-mediated suppression of the soybean cyst nematode

**DOI:** 10.1186/s40168-024-01840-x

**Published:** 2024-07-15

**Authors:** Muzammil Hussain, Peixue Xuan, Yi Xin, Haikun Ma, Yahan Zhou, Shihui Wen, M. Imran Hamid, Tianyu Wan, Jianyang Hu, Yuezhong Li, Seogchan Kang, Xingzhong Liu, Meichun Xiang

**Affiliations:** 1 State Key Laboratory of Mycology, Institute of Microbiology, Chinese Academy of Sciences, Beijing, 100101 China; 2 University of Chinese Academy of Sciences, Beijing, 100049 China; 3 Department of Microbiology, State Key Laboratory of Medicinal Chemical Biology, Key Laboratory of Molecular Microbiology and Technology of the Ministry of Education, Frontiers Science Center for Cell Responses, College of Life Science, Nankai University, Tianjin, 300071 China; 4 Department of Botany and Plant Science, University of California, Riverside, CA 92507 USA; 5 State Key Laboratory of Microbial Technology, Shandong University, Qingdao, 266237 China; 6 Department of Plant Pathology & Environmental Microbiology, The Pennsylvania State University, University Park, PA 16802 USA

**Keywords:** Chitinase, Cyst microbiota, *Heterodera glycines*, Induced resistance, Nematode suppression, Soybean, Suppressive soil

## Abstract

**Background:**

Soybean cyst nematodes (SCN) as animal parasites of plants are not usually interested in killing the host but are rather focused on completing their life cycle to increase population, resulting in substantial yield losses. Remarkably, some agricultural soils after long-term crop monoculture show a significant decline in SCN densities and suppress disease in a sustainable and viable manner. However, relatively little is known about the microbes and mechanisms operating against SCN in such disease-suppressive soils.

**Results:**

Greenhouse experiments showed that suppressive soils (S) collected from two provinces of China and transplantation soils (CS, created by mixing 10% S with 90% conducive soils) suppressed SCN. However, SCN suppressiveness was partially lost or completely abolished when S soils were treated with heat (80 °C) and formalin. Bacterial community analysis revealed that the specific suppression in S and CS was mainly associated with the bacterial phylum Bacteroidetes, specifically due to the enrichment of *Chitinophaga* spp. and *Dyadobacter* sp., in the cysts. SCN cysts colonized by *Chitinophaga* spp. showed dramatically reduced egg hatching, with unrecognizable internal body organization of juveniles inside the eggshell due to chitinase activity. Whereas, *Dyadobacter* sp. cells attached to the surface coat of J2s increased soybean resistance against SCN by triggering the expression of defence-associated genes. The disease-suppressive potential of these bacteria was validated by inoculating them into conducive soil. The *Dyadobacter* strain alone or in combination with *Chitinophaga* strains significantly decreased egg densities after one growing cycle of soybeans. In contrast, *Chitinophaga* strains alone required more than one growing cycle to significantly reduce SCN egg hatching and population density.

**Conclusion:**

This study revealed how soybean monoculture for decades induced microbiota homeostasis, leading to the formation of SCN-suppressive soil. The high relative abundance of antagonistic bacteria in the cyst suppressed the SCN population both directly and indirectly. Because uncontrolled proliferation will likely lead to quick demise due to host population collapse, obligate parasites like SCN may have evolved to modulate virulence/proliferation to balance these conflicting needs.

Video Abstract

**Supplementary Information:**

The online version contains supplementary material available at 10.1186/s40168-024-01840-x.

## Introduction

Continuous monoculture often causes poor crop growth due to changes in soil properties, such as acidification, autotoxin accumulation, increased soilborne pathogen inoculum, and bacterial community dysfunction [1]. This practice has also been documented to induce soil suppressiveness against soilborne pathogens [2]. Disease suppressiveness could be transferred to “disease-conducive soils” by incorporating a small amount (1–10% w/w) of “disease-suppressive soils” [2, 3] but eliminated by heat or biocidal treatment, suggesting that microorganism(s) cause disease suppression and carry soil memory of disease suppressiveness [4]. When faced with severe soilborne disease, plant roots "cry for help" to recruit microbial species antagonistic to the causal pathogen [5, 6]. The resulting disease suppressiveness may dissipate in the absence of disease pressure but will bounce back upon new infections [2]. Advances in sequencing, bioinformatics, and functional genomics have helped identify and quantify soil/plant-associated microorganisms and predict the biology and role of identified taxa using previously characterized species as references [7]. Characterization of disease-suppressive soils using such approaches has uncovered some underlying mechanisms, such as the production of non-ribosomal peptide antibiotics by *Pseudomonas* and *Flavobacterium* in sugar beet-associated suppressive soils [3, 6] and the secretion of a heat-stable antifungal thiopeptide against *Fusarium oxysporum* by *Streptomyces* present in strawberry monoculture soil [8].

**Fig. 1:**
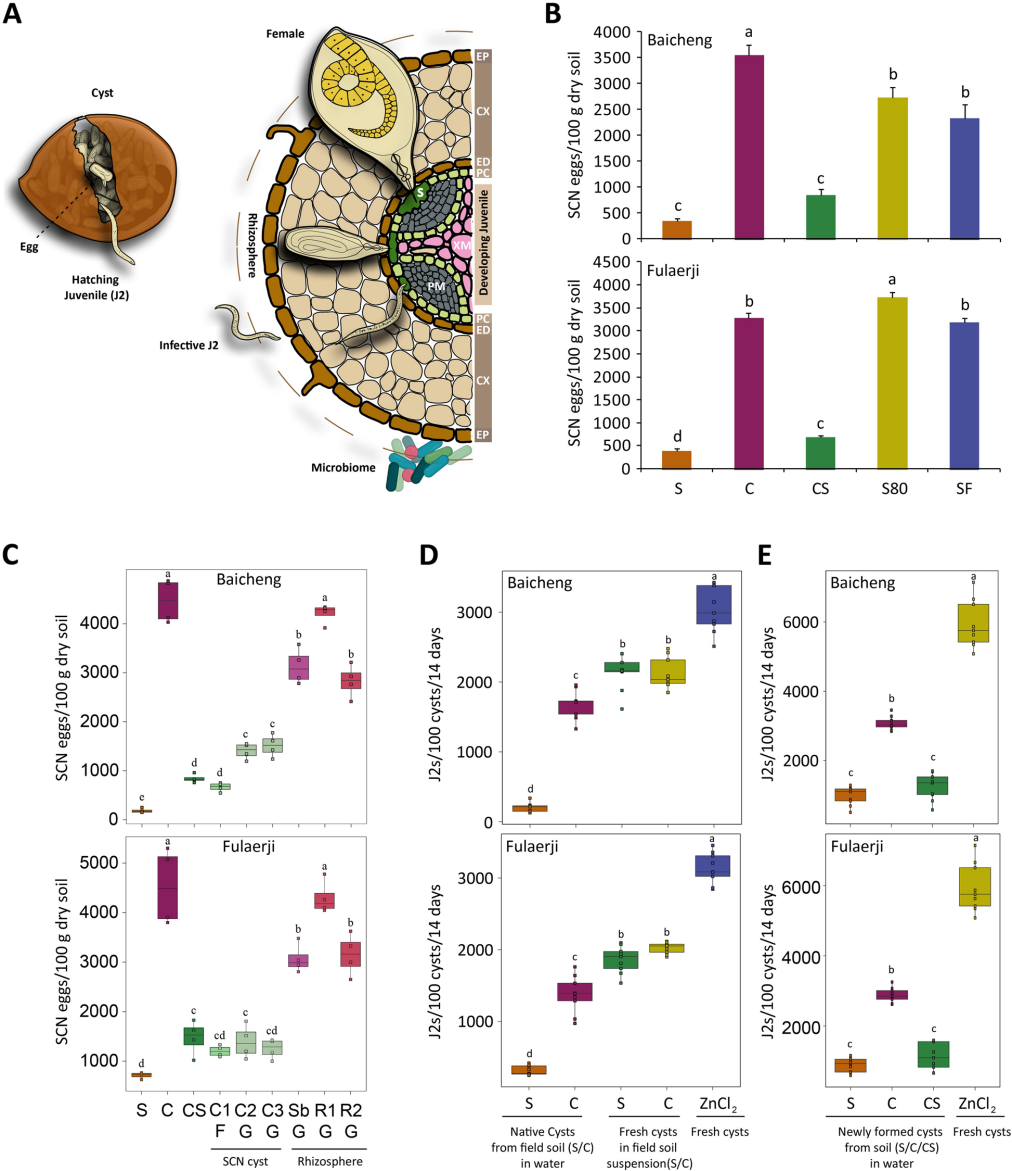
The infection cycle of soybean cyst nematode (SCN), SCN suppressiveness of the field soils used in this study, and how various soil treatments affected SCN suppressiveness. **A** SCN eggs in the cyst hatch to release infective second-stage juvenile (J2) when a host is available. The J2 migrate towards soybean roots, penetrate to reach the vascular cylinder, and stimulate cylinder cells to form the syncytium (S), a feeding site that supports J2 differentiation into an adult nematode. Lemon-shaped adult female protrudes through the root surface and becomes the cyst when the female dies. Mature cysts typically fall off into the surrounding soil and remain dormant until stimulated by root exudates. ED, PC, PM and XM denote endodermis, pericycle, phloem, and xylem, respectively. **B** Egg densities were measured after growing soybean plants for 56 days in field-treated soils inoculated with 1500 eggs per 100 g of dry soil. Different letters denote statistically significant differences according to the LSD test (*p* < 0.05). Suppressive (S) and conducive (C) soils collected from two provinces, transplantation soil (CS; created by mixing 10% S soil with 90% C soil), heat (80 °C)-treated S soil (S80), and S soil fumigated with formalin (SF) were used. **C** Identification of the likely sources of microbiota-suppressing SCN. Treatments of C, S and CS identical to those shown in **B** were included as references. The cyst and soybean rhizosphere treatments were conducted by replacing 10% S in the CS treatment with the following preparations derived from S soils: native cysts isolated from S field soils (C1-F), newly-formed cysts developed on soybean in S soils under greenhouse condition (C2-G) and suspension prepared from C2-G cysts (C3-G). For soybean rhizosphere, soybean seedling grown in S soil for 2 weeks and transplanted in C soil (Sb-G), rhizosphere soil prepared from soybean seedlings grown in S soils and then transfer to C soils (R1-G), and suspension prepared from of soybean rhizosphere and root in S soils and transferred to C soils (R2-G). **D** and **E** The number of J2s hatched after 100 cysts harvested from various soils were placed for 2 weeks in water. A solution of 0.05% ZnCl_2_ was used to stimulate egg hatching. The native cysts used were extracted from the field S and C soils. Fresh cysts referred to the cysts collected after growing soybean for 56 days in autoclaved soil. Newly formed cysts referred to the cysts collected after growing soybean in the soils of S, C, and CS treatments

Like pathogen suppressiveness, the soil suppressiveness of plant parasitic nematodes (PPN), threats to many crops around the world [9], can be induced by long-term monoculture [10, 11]. Because PPN require living hosts to complete their life cycle, they do not kill host plants. However, they can cause severe yield losses, especially when the inoculum level is high, by disrupting root development/function, syphoning off nutrients, and facilitating root infections by soilborne pathogens. Root-knot and cyst nematodes are sedentary endoparasites and damage many crops worldwide [12]. Cyst nematodes produce the cyst, a structure derived from dead female body to keep eggs carrying infective juveniles protected during long-term dormancy (Fig. 1A). Previous studies have reported suppressive soils against cyst nematodes, including *Heterodera avenae* in wheat [13], *H. schachtii* in sugar beet [14], *Globodera rostochiensis* and *G. pallida* in potatoes [15–17], and *H. glycines* in soybean [10, 11]. Some fungal species, such as *Nematophthora gynophila* and *Pochonia chlamydosporia* in *H. avenae*-suppressive soils [13], and *Dactylella oviparasitica* in sugar beet cyst nematode-suppressive soils, have been shown to suppress specific nematodes [14]. Interestingly, *H. schachtii* cysts infested with *D*. *oviparasitica* in suppressive soils could transfer suppressiveness into *H. schachtii*-infested conducive soils [14].

With an annual production of 330 million tons, soybean is a vital food/feed crop that is also widely used for biofuel production and nitrogen fixation [18]. The soybean cyst nematode (SCN, *H. glycines*) is one of the most devastating threats to soybean production, annually costing > 2 billion US dollars due to yield loss in the USA [19]. In China, SCN is distributed across 22 provinces [20], causing yield losses equivalent to ~ 120 million US dollars per year [21]. Soybean monoculture has been shown to create SCN-suppressive soils in both China and the USA [10, 11]. Previous analyses have identified diverse microbial taxa associated with SCN cysts and the soybean rhizosphere and endosphere [22–25]. Despite extensive research on SCN-suppressive soils, the mechanism underpinning SCN suppression remains poorly understood. We previously reported that upon SCN infestation, the relative abundance of some bacterial taxa in bulk soils, the soybean rhizosphere and endosphere, and the cysts increased [25]. However, which taxa are crucial for SCN suppressiveness and how they suppress SCN remained elusive. Since sugar beet cyst nematode suppressiveness could be transferred by incorporating its cysts formed in suppressive soils [14], and that the cyst bacterial community was established via consecutive selection of taxa from the root endosphere [25], we hypothesized that cyst-associated microorganisms are critical for SCN suppressiveness. To test this hypothesis, we performed growth room pot experiments with soybean grown in SCN-suppressive (S) and conducive (C), transplantation (CS, created by mixing 10% S soil with 90% C soil), and heat or formalin-treated S soils. Our specific objectives were to (a) evaluate whether different soils and cysts formed in these soils suppress SCN, (b) comparative analysis of the composition of bacterial communities associated with SCN cysts formed in these soil treatments; (c) isolation and identification of candidate bacterial taxa from SCN cysts, (d) verification of the involvement of these bacteria in disease suppressiveness, and (e) elucidation of the mechanisms employed by cyst-enriched bacteria to suppress SCN.

## Results

### Contribution of SCN cysts to disease suppressiveness

SCN suppressive and conducive soils collected from two regions in China, Baicheng (BC) in Jilin Province and Fulaerji (FL) in Heilongjian Province, were used to investigate how SCN suppressiveness forms and work. The S soils from BC (45 years of monoculture) and FL (37 years of monoculture) harbour significantly fewer SCN eggs than the C soils collected in nearby fields with only 3 years of monoculture (Fig. S1A). Their physical and chemical characteristics were similar (Table S1). Soybean plants grew slightly better in the S soils than the C soils, but the difference was insignificant (Fig. S1B). When soybean plants were grown for 56 days in the S, C, and CS soils inoculated with SCN eggs (1500 eggs per 100 g of dry soil), the resulting egg densities in the S and CS soils were significantly lower than those in the other soils (Fig. 1B and C; Fig. S1C), confirming SCN suppressiveness. Heat treatment at 80 °C (S80) and fumigation resulted in a partial to complete loss of SCN suppressiveness (Fig. 1B and C).

SCN suppressiveness of the soils collected in BC and FL appeared primarily due to the cyst-associated microbiota rather than the rhizosphere microbiota (Fig. S2A and S2B). To determine whether SCN suppression is caused by cyst microbiota or rhizosphere microbiota, instead of using 10% S soil to make CS, we designed three different regimens each for SCN cysts and soybean rhizosphere to differentiate their involvement in disease suppression. The cyst treatments included SCN native cysts isolated from S soils and transferred to C soils (C1-F), newly-formed cysts collected from soybean roots after 56 days of soybean grown in S soils under greenhouse condition and transferred to C soils (C2-G), and a microbiota suspension prepared by ground C2-G cysts and transferred to C soils (C3-G). Similarly, the three soybean rhizosphere treatments included the transfer of soybean seedlings grown in S soils for 2 weeks and transplant to C soils (Sb-G), rhizosphere soil collected from soybean seedlings grown in S soils for 2 weeks and then transferred to C soils (R1-G), and a microbiota suspension prepared from rhizosphere and root of soybean seedling from S soils were transferred to C soils (R2-G). All the cyst treatments significantly lowered egg densities when mixed with 90% C soils, resulting in densities as low as those observed in CS soils created using the soils collected at BC and FL (Fig. 1C). However, the rhizosphere treatments of 90% C soils were not as effective as the cyst treatments in reducing egg densities (Fig. 1C).

Egg-hatching rates of the cysts extracted from the field S soils were significantly lower than those from the field C soils. In contrast, soil extracts prepared from the S and C soils did not significantly affect egg hatching of the freshly prepared cysts using an autoclaved soil (Fig. 1D). However, when the cysts formed in autoclaved soils were placed into the S and C soils for 2 weeks, the cysts recovered from the S soils showed lower egg-hatching rates than those from the C soils (Fig. 1E), suggesting that the cysts acquired some microbiota inhibitory to egg hatching and that SCN suppressiveness could be transferred to different soils by the cysts colonized by such microbiota.

### Identification of candidate bacterial species involved in SCN suppression

**Fig. 2:**
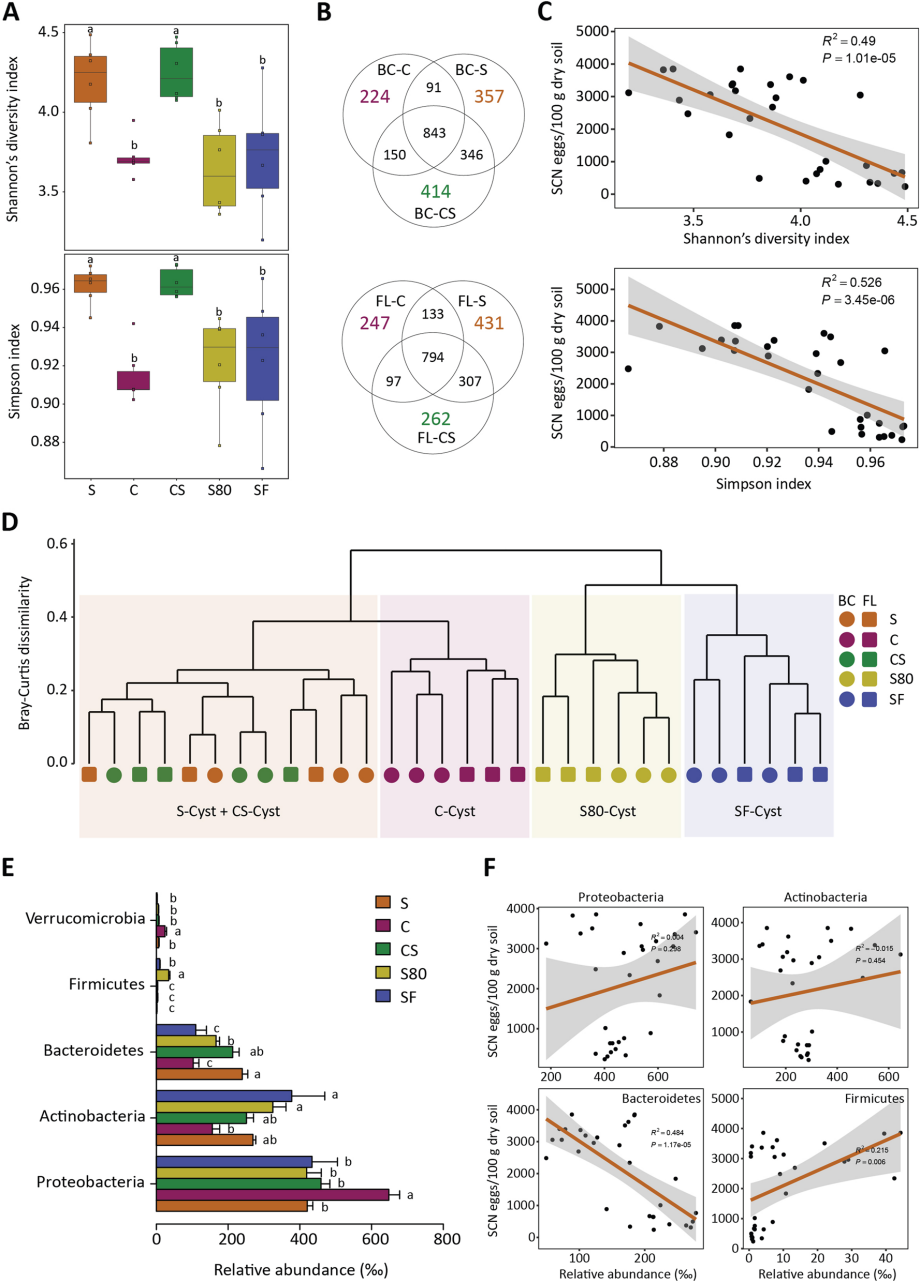
Analyses of cyst-associated bacterial communities to identify candidate taxa for SCN suppressiveness. **A** Within-sample diversity (α-diversity) of bacteria associated with the cysts formed in the S, C, and CS soils and the S soil treated with heat (S80) or formalin (SF). The horizontal bar within each box indicates median value. The top and bottom of boxes denote the 75th and 25.^th^ percentiles, respectively. Upper and lower whiskers extend 1.5 × the interquartile range from the upper and lower edges, respectively. All outliers are plotted as individual points. Different letters indicate statistically significant differences according to the LSD test (*p* < 0.05). **B** Numbers of unique and shared OTUs associated with cyst samples collected from the C, S and CS soils. **C** Linear regression relationship between cyst bacterial diversity and egg density (eggs/100 g of dry soil). The regression line is denoted by brown, and the shaded region indicates the confidence interval (geom_smooth function, method = lm). **D** Cluster analysis based on Bray–Curtis dissimilarity indicates the clear separation of bacterial communities inhabiting the cysts formed in the S and CS soils from those formed in the C, S80, and SF soils. OTUs with RA > 1‰ in at least one sample were included in the analysis. **E** Average RA of the most dominant taxa in the cysts formed in the S, C, CS, S80, and SF soils. Only taxa with RA > 1‰ in at least one sample were included in the analysis. Different letters above the bars indicate significant differences at the *P* < 0.05 level, according to the LSD test. **F** Linear regression relationship between four bacterial phyla associated with cysts and the egg density in the soil. The regression line is denoted by brown, and the shaded region corresponds to the confidence interval (geom_smooth function, method = lm)

To prepare for testing the hypothesis that cyst-associated bacteria confer SCN suppressiveness, we analysed and compared the microbiome of the cysts formed in S, C, CS, S80 and SF soils. In total, > 1.4 million high-quality-filtered reads, corresponding to 3073 bacterial OTUs (> 97% sequence identity), were obtained from 30 samples. The α-diversities, including Shannon’s diversity index and Simpson index of cyst bacterial communities, were higher in the S and CS treatments than in the C, S80 and SF treatments (Fig. 2A; Fig. S3A). Cysts formed in the S and CS soils harboured more shared bacterial OTUs than those formed in the S and C or CS and C soils (Fig. 2B). Significantly negative correlation was observed between the cyst bacterial diversity and SCN egg densities (*P* = 1.01e − 05, *P* = 3.45e − 06; Fig. 2C).

A Bray–Curtis dissimilarity analysis showed that the cyst-associated bacterial communities at both soil sampling locations could be separated into two main clades, one corresponding to the S, CS and C soils and the other for the S80 and SF soils, in the cluster dendrogram, indicating that heat- and formalin-treatments had a significant impact on the assembly of cyst bacterial microbiota. Those corresponding to S and CS form one subclade, and those for C form another subclade, consistent with the degree of SCN suppressiveness (Fig. 2D; Fig. S3B). Overall, these results imply that the bacterial microbiota associated with SCN suppressiveness could be transferred to C soils by adding a small amount of S soils and the disease-suppressive bacteria can effectively colonize and proliferate in newly formed cysts.

Analysis of the relative abundance (RA) of different phyla suggested Bacteroidetes as the phylum associated with SCN suppressiveness. The cysts formed in the S and CS soils have significantly greater RA of Bacteroidetes and family Chitinophagaceae than those formed in C soils (Fig. 2E; Fig. S4A, S4B, S5A and S5B), and their RA was reduced when the S soils were treated with heat or formalin. Linear relationship analysis showed a significant negative correlation between the RA of Bacteroidetes and egg density (*P* = 1.17e − 05; Fig. 2F), supporting their role in SCN suppressiveness. The RA of Actinobacteria inhabiting the cysts formed in S and CS was also higher than that in C, but the difference was not statistically significant compared to the other treatments (LSD test).

**Fig. 3:**
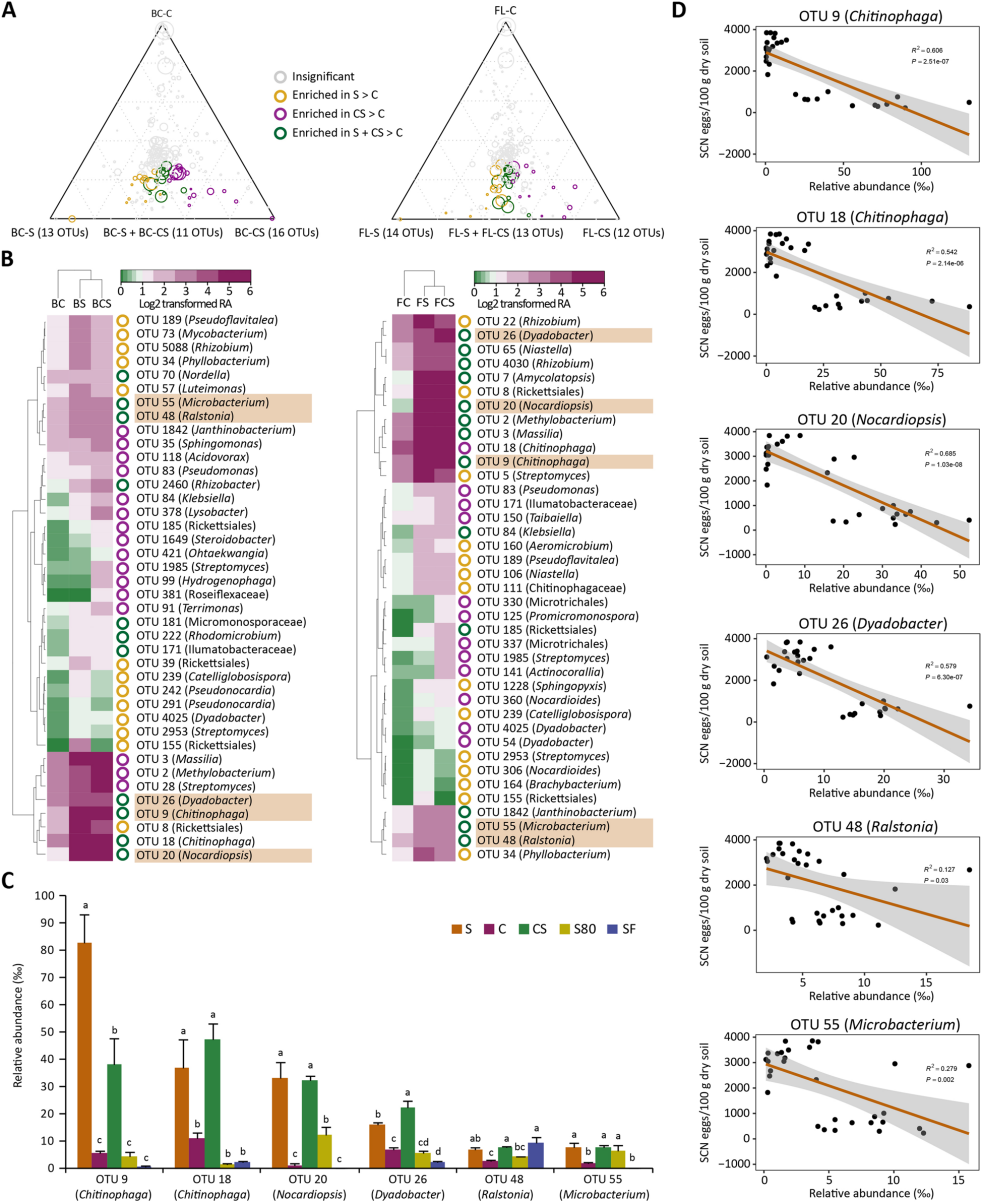
Identification of candidate bacterial OTUs associated with SCN suppressiveness in two different locations. **A** Ternary plots show OTUs significantly enriched in the cysts formed in the S and CS soils compared to those formed in the C soils of BC and FL. The colour and size of the circle denote the samples in which individual OTUs are enriched and their relative abundance (RA), respectively. Golden (OTUs significantly enriched in those formed in S compared to C; S > C OTUs); Pink (OTUs significantly enriched in those formed in CS compared to C; CS > C OTUs); Green (OTUs enriched in those formed in both S and CS compared to C; S + CS > C OTUs); Grey (those that were not enriched in any samples). The position of each circle is determined by the contribution of the indicated compartments to the total RA. Only the OTUs with RA > 1‰ in at least one sample were included in the analysis. **B** Heatmaps showing RAs of the OTUs significantly enriched in the cysts formed in the S (golden circle), CS (pink circle) and both S and CS (green circle) soils. The OTUs highlighted were enriched in cysts formed in both the S and CS soils from Baicheng (BC) and Fulaerji (FL). **C** The RA of six OTUs enriched in cysts formed in S and CS compared to the other three treatments. Different letters above the bars indicate significant differences at the *P* < 0.05 level, according to the LSD test. **D** Linear regression relationship between the RA of *Chitinophaga* (OTU9 and OTU 18), *Nocardiopsis* (OTU20), *Dyadobacter* (OTU26), *Ralstonia* (OTU48), and *Microbacterium* (OTU55) and the egg density (eggs/100 g dry soil). The regression line is denoted by brown, and the shaded region represents the confidence interval (geom_smooth function, method = lm)

We identified OTUs enriched in the cysts formed in the S soils from BS and FL and the CS soils derived from these S soils. For the cysts formed in the S soils of BC and FL, 13 and 14 OTUs, respectively, were significantly enriched. For those formed in the CS soils, 16 (BC) and 12 (FL) OTUs were significantly enriched. In contrast, 11 (BC) and 13 (FL) OTUs were enriched in both S and CS soils. Among these enriched OTUs, only 5 were commonly present in those formed in both the S and CS soils (Fig. 3A). One OTU (OTU18) was enriched in cysts formed in the S (BC) and CS (both BC and FL) soils (Fig. 3B), and its presence significantly correlated with reduced SCN egg density. Six OTUs potentially associated with SCN suppression are *Chitinophaga* (OTU9 and OTU18) and *Dyadobacter* (OTU26) of Bacteroidetes, *Nocardiopsis* (OTU20) and *Microbacterium* (OTU55) of Actinobacteria, and *Ralstonia* (OTU48) of Proteobacteria.

Further analysis of these 6 OTUs after different treatments revealed that the RA of *Chitinophaga* (OTU9 and OTU18), *Dyadobacter* (OTU26) and *Nocardiopsis* (OTU20) was significantly higher in the S and CS soils (Fig. 3C) and negatively correlated with the egg density (Fig. 3D). The RAs of *Ralstonia* (OTU48) after SF and S80 treatments and *Microbacterium* (OTU55) after S80 treatment were not significantly different from those of S (Fig. 3C). *Chitinophaga* (OTU9 and OTU18), *Dyadobacter* (OTU26) and *Nocardiopsis* (OTU20) were highly sensitive to SF and S80 treatments (Fig. 3C). In addition, the S80 and SF treatments significantly shifted the RA of several S OTUs (Fig. S6). Because *Chitinophaga* and *Dyadobacter* had the highest RA in the cysts formed in the S soils, and their RAs were significantly correlated with reduced nematode egg density, we next investigated if whether these two Bacteroidetes suppress SCN by isolating strains belonging to these taxa.

### *Chitinophaga* and *Dyadobacter* employ distinct mechanisms to suppress SCN

**Fig. 4:**
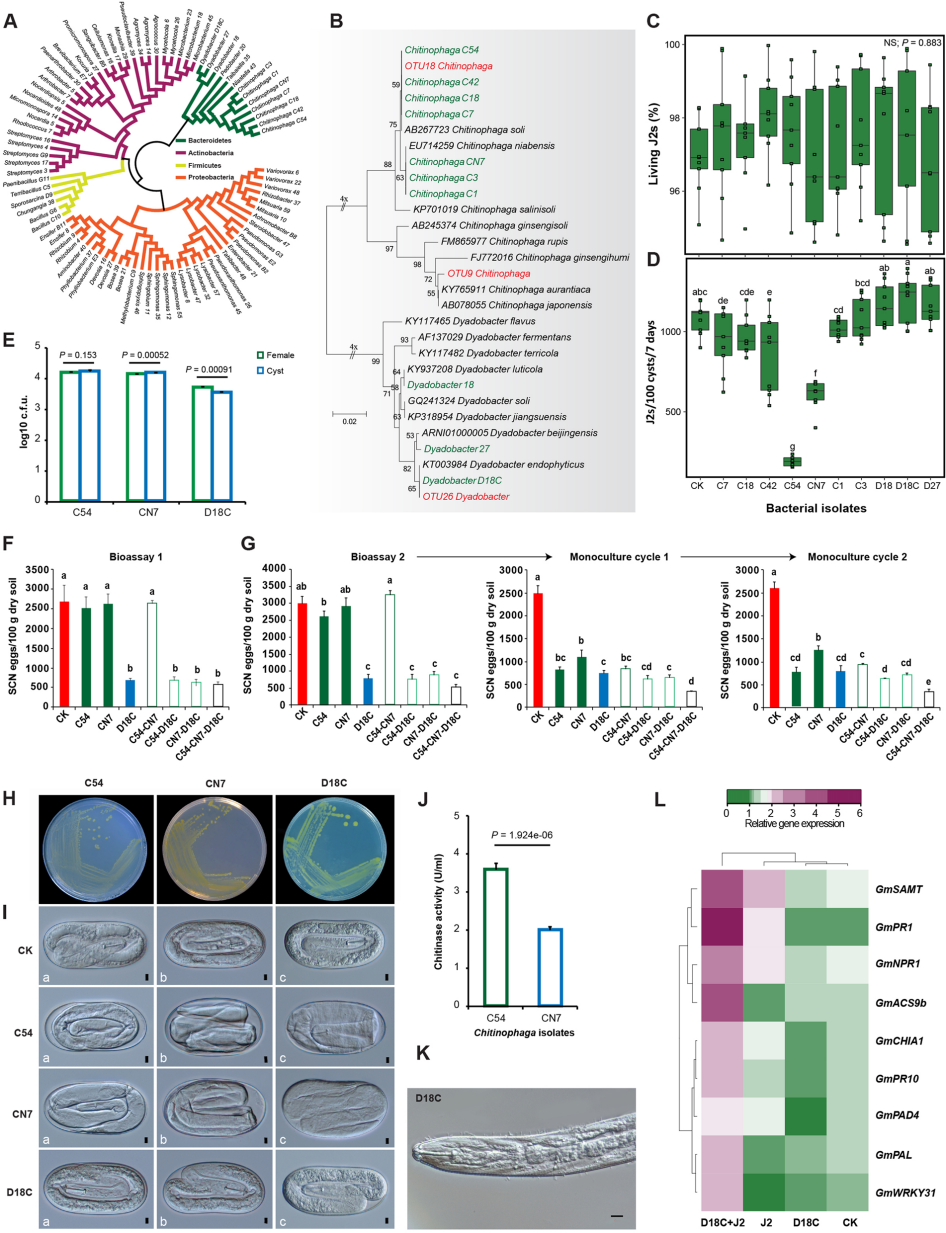
Analyses of how *Chitinophaga* and *Dyadobacter* isolates affect SCN individually and in combinations. **A** Cladogram showing the phylogenetic relationship among the cultured strains (identified using their 16S rRNA sequences). **B** Phylogenetic tree showing the relationship of OTU9, OTU18 and OTU26 with previously characterized *Chitinophaga* and *Dyadobacter* spp. Black and green denote those retrieved from GenBank and the isolates from this study, respectively. Two OTUs corresponding to *Chitinophaga* and one OTU corresponding to *Dyadobacter* (red) were included in this analysis. **C** The effect of *Chitinophaga* (C7, C18, C42, C54, CN, C1, C3) and *Dyadobacter* (D18, D18C, D27) isolates on the viability of SCN J2s (percentage of living J2s after coincubation). **D** The number of J2s hatched from 100 cysts 7 days after incubation with each isolate. Water was used as the control (CK). **E** The numbers of *Chitinophaga* (C54 and CN7) and *Dyadobacter* (D18C) cells per SCN female and cyst were quantified. The females and cysts collected after 8 weeks of soybean growth in autoclaved C soil infested with SCN eggs were used. **F** Egg densities were measured 56 days after growing soybean plants in the C soil amended with SCN eggs (1500/100 g of dry soil) and C54, CN7, D18C, C54-CN7, C54-D18C, CN7-D18C, and C54-CN7-D18C. Two independent assays were performed. The total number of inoculated bacterial cells was 10^7^ CFU/g of dry soil, regardless the number of isolates used. **G** After measuring egg densities as described in (**F**), two more cycles of soybean plant growth for 56 days in the same soils were performed without adding new bacterial cells. Egg density was measured after each cycle. Error bar denotes the standard error of the mean. Different letters indicate statistically significant differences between treatments as determined by one-way ANOVA with the LSD test (*P* < 0.05). **H** Colony morphology of *Chitinophaga* (C54 and CN7) and *Dyadobacter* (D18C) isolates on R2A medium. **I** Microscopic observation of eggs carrying J1 after incubating the cysts with *Chitinophaga* (C54 and CN7) and *Dyadobacter* (D18C) in autoclaved C soil. Water served as the control (CK). **J** Chitinase activity of *Chitinophaga* isolates C54 and CN7 in the presence of SCN eggs. **K** J2 with attached *Dyadobacter* isolate D18C. Scale bar = 5 µm. **L** Heatmap showing relative expression levels of selected soybean defence-related genes involved in the salicylic acid, jasmonic acid and ethylene signalling pathway. *Gm*SAMT, salicylic acid methyltransferase; *Gm*PR1, pathogenesis related-1; *Gm*NPR1, non-expresser of pathogenesis related-1 gene; *Gm*ACS9b, 1-Aminocyclopropane-1-carboxylic acid synthase; *Gm*CHIA1, chitinase class I; *Gm*PR10, pathogenesis related-10; *Gm*PAD4, phytoalexin deficient 4; *Gm*PAL, phenylalanine ammonia lyase; *Gm*WRKY31. The data were normalized using the reference gene *GmActin*

Bacterial species belonging to Proteobacteria, Actinobacteria, Bacteroidetes, and Firmicutes were isolated from cysts formed in the S soils (Fig. 4A). A phylogenetic analysis of several *Chitinophaga* and *Dyadobacter* isolates, identified using their 16S rRNA gene sequences, revealed that the *Chitinophaga* isolates clustered with two species (*C. soli* and *C. niabensis*) and the *Dyadobacter* isolates clustered with *D. luticola*, *D. beijingensis*, and *D. endophyticus*. The 16S rRNA sequences of *Chitinophaga* C7, C18, C42, C54 and *Dyadobacter* D18C were 100% identical to OTU18 and OTU26, respectively (Fig. 4B).

The effect of *Chitinophaga* and *Dyadobacter* isolates on J2 mortality and egg hatching was first investigated. When freshly prepared J2s were incubated with individual isolates, most of them (~ 97%) remained alive 7 days post inoculation, indicating that they did not cause J2 mortality (Fig. 4C). However, when freshly prepared cysts were incubated with these isolates in autoclaved soils, *Chitinophaga* isolates C54 and CN7, but not *Dyadobacter* isolates, significantly reduced egg hatching (Fig. 4D). *Chitinophaga* abundance was significantly higher in cysts than in soil (Fig. S7). All three isolates (C54, CN7 and D18C) were able to colonize SCN females and cysts (Fig. 4E).

Soybean plants were grown for 56 days in the C soil mixed with extra eggs (1500/100 g of dry soil) and *Chitinophaga* (C54 and CN7) and *Dyadobacter* (D18C), individually and in combination. SCN could complete two life cycles during this period. Only D18C, D18C-C54, D18C-CN7, and D18C-C54-CN7 significantly decreased the egg density (Fig. 4F), suggesting that CN7 and C54 alone may require more than one growth cycle to reduce egg hatching and vitality. To test this hypothesis, we grew new soybean plants in the same pots twice (56 days for each cycle) without additional treatments. Egg density was significantly reduced by all treatments after the second and third cycles (Fig. 4G), supporting the involvement of both *Chitinophaga* strains in SCN suppression.

Microscopic observation of the eggs in cysts treated with these three isolates (Fig. 4H), indicated that *Chitinophaga* C54 and CN7 caused severe malformations of first-stage juveniles (J1s) within eggs (Fig. 4I). Their body organization was abnormal and eventually deteriorated completely. Both isolates significantly suppressed egg hatching, comparable to that of the cysts formed in the S soils (Fig. 4D). The glycosyl hydrolase (GH18) family of chitinases in *Chitinophaga* isolated from disease-suppressive soils have been shown to contribute to suppressing fungal pathogens (Carrion et al., 2019). Consistent with this, both *Chitinophaga* isolates CN7 and C54 produced chitinases in the presence of SCN eggs, which supported their role in SCN-suppressive activity (Fig. 4J). We then sequenced the CN7 and C54 genomes using PacBio sequencing (Fig. S8 and S9). Based on the average nucleotide identity, the close relatives of CN7 and C54 were *C. niabensis* and *C. soli*, respectively. To further confirm the involvement of the chitinases in SCN suppression, we identified three putative GH18 family genes in the CN7 genome (Chpbs_4014, Chpbs_4904 and Chpbs_5684) and determined their expression levels using qRT-PCR. They exhibited distinct patterns of expression in the presence of SCN eggs, with the expression of Chpbs_5684 significantly increasing at 24 and 48 h after applying CN7 (Fig. S10A). The expression of Chpbs_4014 did not change, whereas the expression level of Chpbs_4904 appeared to slightly increase at 24 h, but not at 48 h. We then produced chitinases encoded by these genes in *Escherichia coli* to test their activity against SCN (Fig. S10B and S10C). Chitinases in the culture filtrate of CN7 and those partially purified using *E. coli* were sufficient to deform juveniles in eggs and stop egg hatching.

*Dyadobacter* (D18C) cells were found attached to the surface coat of J2s hatched under axenic conditions (Fig. 4K). The absence of a direct negative effect of D18C on nematode suggested that this strain might indirectly suppress SCN by affecting soybean plants. To test this hypothesis, we inoculated soybean plants with SCN J2s, D18C, and J2s with attached D18C and quantified the expression levels of selected soybean defence-related genes. The expression of three genes under the control of the salicylic acid signalling pathway (*Gm*SAMT *Gm*PR1 and *Gm*NPR1) was significantly up-regulated by the inoculation of SCN J2 relative to those treated with water and D18C. In contrast, only J2 colonized by D18C significantly induced the expression of defence-related genes *Gm*SAMT, *Gm*PR1, *Gm*NPR1, *Gm*ACS9b, *Gm*CHIA1, *Gm*PR10, *Gm*PAD4, *Gm*PAL, and *Gm*WRKY31 involved in the salicylic acid, jasmonic acid and ethylene pathways at different levels (Fig. 4L).

## Discussion

Synthetic pesticides have helped limit crop losses caused by diseases and pest infestations but incur increasingly unsustainable environmental and ecological costs. Rapid advances in understanding how biotic and abiotic factors affect plant growth and health and their underlying mechanisms have greatly facilitated the development of alternative strategies for crop protection [7]; however, many critical knowledge deficiencies still exist. Here, we addressed one such deficiency, how soil suppressiveness of SCN forms and works, to support the effective management of SCN and other PPN. Because SCN persists once established, available control strategies, such as crop rotation and planting SCN-resistant varieties, target to suppress its population to a level that does not cause significant yield loss. Our study offers new insights that can be harnessed to augment existing strategies and develop new ones.

Continuous cultivation of soybean plants susceptible to SCN initially increases the density of SCN, but the population declines significantly after several years [10, 11]. Induced soil suppressiveness is not unique to the soybean-SCN system and has been observed in other cyst nematode-crop systems, including *Heterodera avenae*–cereals [13], *H. schachtii*-sugar beets [14], and *Globodera pallida*/*G. rostochiensis*-potato [15–17]. Similar to pathogen-suppressive soils [2], soil suppression of PPN is characterized by the ability of resident microbial communities to reduce nematode populations [26]. Metabarcoding analyses of microbial communities associated with different parts of diverse plants and soils have helped identify taxa correlated with disease suppressiveness [3, 6, 8, 27, 28]. However, in most cases of plant pathogen-suppressive soils, the studies are descriptive, candidate taxa have not been identified at the species level, and their involvement in suppression has not been validated [29–31]. Accordingly, the microbial traits that drive pathogen suppression remain largely mostly unknown, with a few exceptions. For example, genes encoding non-ribosomal peptide synthetases and polyketide synthases are crucial for root disease suppression by *Chitinophaga* and *Flavobacterium* enriched in sugar beet roots, suggesting the involvement of secondary metabolites in pathogen suppression [6]. For cyst nematode-suppressive soils, the focus has been on identifying fungal species associated with disease suppressiveness [14, 17, 22, 32–36]. Analyses of fungal communities inhabiting *H. schachtii* cysts identified *Hyalorbilia oviparasitica* (formerly *Dactylella oviparasitica*), *Fusarium oxysporum*, and *Lycoperdon* spp. as potential taxa underlying suppressiveness, and subsequent analyses confirmed the involvement of *H. oviparasitica* in suppressiveness [32]. Interestingly, *H. schachtii* cysts infested with *H. oviparasitica* in suppressive soils could transfer suppressiveness to *H. schachtii*-infested conducive soil [14].

SCN cysts likely harbour diverse microorganisms because females encounter soil and root tissue-associated microbiota as they migrate towards roots, penetrate roots, and differentiate to form cysts [25]. A previous study estimated 2.6 ± 0.5 × 10^5^ bacteria inhabiting a SCN cyst [37]. Such bacteria likely affect the viability and fitness of nematodes, but the taxa linked to nematode suppressiveness remain unknown. We observed that SCN suppressiveness could be established and transferred by bacteria isolated from cysts formed in S soils (Fig. 1C). This observation, the transfer of SCN suppressiveness by incorporating cysts formed in S soils [14], and previous studies showing the critical role of bacteria in fungal pathogen-suppressive soils led us to analyse cyst-associated bacterial taxa to identify ones closely linked to SCN suppressiveness. This analysis (Figs. 3 and 4) revealed that Bacteroidetes *Chitinophaga* and *Dyadobacter*, enriched in the cysts formed in S soils confer and transmit SCN suppressiveness. Isolation of multiple strains belonging to *Chitinophaga* and *Dyadobacter* allowed us to validate their role in SCN suppression by inoculating them into C soils and to characterize their mechanism of SCN suppression.

**Fig. 5:**
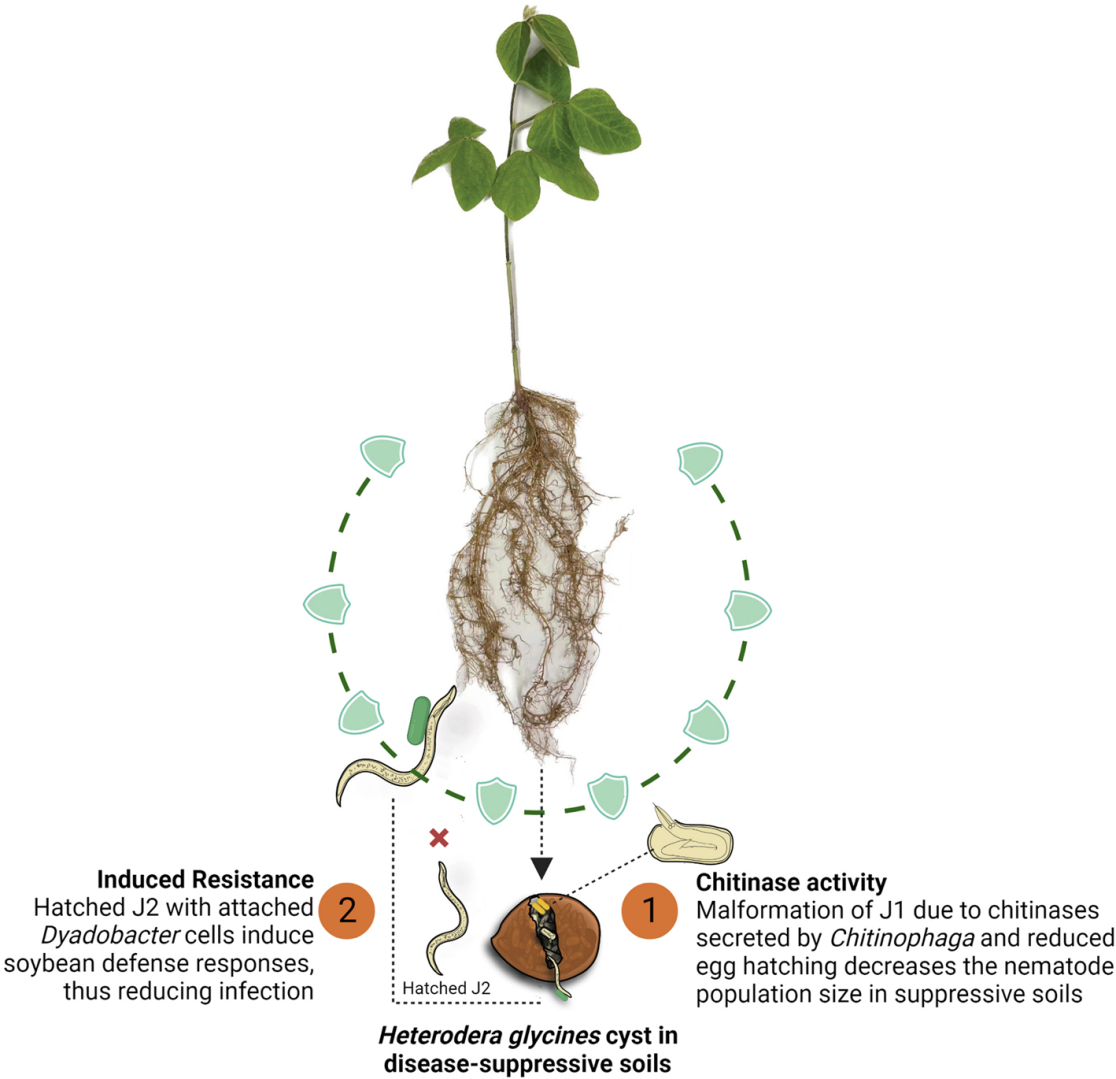
Conceptual model illustrating the mechanism of SCN suppression in suppressive soils. Two cyst-associated bacteria employ distinct mechanisms to suppress SCN. (1) Enrichment of *Chitinophaga* in cysts causes malformation of first-stage juveniles presumably due to their chitinases, significantly reducing viable J2s. (2) Enrichment of *Dyadobacter* in cysts leads to its attachment to the surface coat of hatching J2s, and such J2s induce resistance in soybean by increasing defence-related gene expression, thereby reducing plant infection

Members of *Chitinophaga* are chitin-decomposers and were found to be enriched in the rhizosphere of wheat upon long-term monoculture and infection by *Rhizoctonia solani*, a fungal pathogen of diverse plants [38]. *Chitinophaga* is one of the two key taxa associated with soil suppressiveness of *R. solani* developed after long-term monoculture of sugar beet, and their chitinase activity seems critical for the suppression [6]. Our previous study revealed that the bacterial community in SCN cysts was established because of the consecutive selection of microbial taxa from the soybean root endosphere and that *Chitinophaga* was highly abundant in the cyst, followed by the root endosphere and rhizosphere in suppressive soils (Fig. S7C) [25]. Chitin is an essential element for the fungal cell wall and the nematode eggshell and body, which is why *Chitinophaga* preferentially colonize SCN cyst from the root endosphere and reduces egg hatching (Fig. 4D) and caused morphological defects in the first stage juveniles inside the eggs (Figs. 4I and 5). *Chitinophaga* enrichment and activity against parasitic nematodes and fungal pathogens in disease-suppressive soils suggest that they suppress diverse pests and pathogens. *Dyadobacter* was identified as a taxon potentially involved in suppressing *Fusarium* banana wilt [39], suggesting that it also is involved in suppressing multiple pathogens and pests. Three *Dyadobacter* isolates did not inhibit SCN egg hatching (Fig. 4D). However, isolate D18C attached to the surface coat of hatched J2s (Fig. 4K) strongly induced defence-related gene expression of in soybean plants (Fig. 4L), suggesting that *Dyadobacter* indirectly suppresses SCN by inducing soybean defence against SCN during the initial stage of infection (Fig. 5). It has been previously demonstrated that only a specific subset of soil microorganisms attach to the surface coat of nematodes [40–42]. The carbohydrate-rich protein layer over the nematode epicuticle has been identified as the main area for microbial attachment [43]. The interaction between non-parasitic bacteria and plant root surfaces is lectin-specific, which may results in the recognition of nematodes by plants [44]. Thus, some microorganisms attached to the surface coat of nematode juveniles use them as carriers to the plant roots and induce resistance in plants [45, 46]. Our data suggest that soybean plants have evolved to recognize some of such bacteria.

Our results raised several questions, including why and how long-term monoculture enriches *Chitinophaga* and *Dyadobacter* in SCN cysts. Did SCN evolve to modulate its virulence/proliferation by recruiting specific microbial taxa to balance two conflicting needs (proliferation vs. preventing severe host dysfunction/collapse)? SCN, an obligate pest with limited hosts, needs to balance these needs because uncontrolled proliferation/virulence likely causes a severe fitness penalty. Alternatively, is this driven by an unknown mechanism that soybean has evolved to reduce pest population? Related questions include which factors are critical for the enrichment of *Chitinophaga* and *Dyadobacter*, if these taxa are involved in inducing SCN suppressiveness in diverse fields (as both taxa were enriched in two agricultural fields at distinct geographical location within China) or other taxa/mechanisms drive the suppressiveness in different areas or field conditions, whether *Chitinophaga* and *Dyadobacter* confer suppressiveness against different cyst nematodes affecting other crops, and how soybean plants recognize *Dyadobacter* associated with J2s to activate defence responses. Answers from these questions, along with *Chitinophaga* and *Dyadobacter* isolates that effectively suppress SCN, will help develop novel strategies for controlling SCN and potentially other parasitic nematodes (e.g. modifying soil ecosystems by incorporating formulated *Chitinophaga* and *Dyadobacter* isolates as biocontrol agents, judiciously deploying cultural practices to promote or speed up their SCN colonization/enrichment).

## Conclusions

Our study showed that the specific suppression of SCN in two long-term soybean monoculture soils was due to the enrichment of disease-suppressive bacteria in nematode cysts. The cyst microbiome presented high bacterial diversity and a unique subset of microbes capable of transmitting nematode suppressiveness to conducive soil environments. Specifically, the cyst-enriched bacteria *Chitinophaga* and *Dyadobacter* employed two distinct mechanisms to protect soybean against SCN: (a) malformation of J1 in eggshell due to chitinases secreted by *Chitinophaga* strains and (b) the *Dyadobacter* strain induced soybean defence responses, thus reducing infection. Our findings not only present a compelling case for the trade-off wherein SCN cyst-enriched microbes inhibit nematode proliferation both directly and indirectly, but also shed light on the potential discovery of specific microbial consortia from suppressive soils to develop synthetic microbial communities for targeting plant-parasitic nematodes.

## Methods

### Soil collection and preparation

SCN suppressive soils were first identified in northeastern China by Sun and Liu [10]. The S soils used in the current study were collected from two fields in this region, Baicheng of Jilin Province (BC; N 45°37′ E 122°47′) and Fulaerji of Heilongjian Province (FL; N 47°20′ E 123°62′) with 45 and 37 years of soybean monoculture, respectively, as previously described [24]. The C soils were collected from two fields, located near the S soil collection sites, with only three years of soybean monoculture. Soil cores (depth of 0–30 cm), including soybean roots, were sampled from the rows of plants in a zigzag pattern at the time of crop harvest, and five random sites in each field were sampled. All soil cores from each field were mixed thoroughly and sieved through a 2-mm mesh to remove plant debris and stones.

### Analysis of soil physicochemical properties

Selected physical and chemical properties of the S soils from BC and FL are listed in Table S1. Advanced Standard Technical services (Beijing, China) analysed the physicochemical properties of the soils. Total N and P were measured using an AA3 HR AutoAnalyzer (SEAL Analytical) following the procedures provided by the manufacturer. The data were viewed and analysed using Windows-based AACE software (SEAL Analytical).

### Isolation of SCN cysts and egg density measurement

Soils containing SCN cysts were washed with a vigorously applied water stream through an 850 µm aperture sieve onto a 250 µm aperture sieve and extracted by centrifugation in 76% (w/v) sucrose solution at 2500 rpm for 6 min [25]. Eggs were released by breaking the cysts in a 40 ml glass tissue grinder (Fisher catalogue No. 08–414-10D). The eggs were separated from the debris by centrifugation in a 35% (w/v) sucrose solution for 5 min at 2500 rpm and collected from a 25 µm aperture sieve [47]. The resulting egg samples were dispensed into 12-well tissue culture plates (Nest Biotechnology) and counted using an inverted microscope (Olympus CK40) to calculate egg density in 100 g of dry soil.

### Growth chamber evaluation of SCN suppressiveness

We evaluated the SCN suppressiveness of the following soil samples: (i) S soils, (ii) C soils, (iii) transfer soils prepared by mixing 10% S and 90% C soils (CS), (iv) S soils incubated at 80 °C for 1 h (S80), and (v) S soils treated with formalin (SF). For the heat treatment, a 1000 mL glass flask containing 500 g of each S soil (with an adjusted moisture content of approximately 10%) was placed in a water bath set at 80 °C for 1 h. For formalin treatment, 1.5 kg of each S soil was thoroughly mixed with formalin (3.8 mL 40% formaldehyde per kg of soil) in a 4.5 L plastic bag [11].

We multiplied SCN and produced cysts using the susceptible soybean variety ‘Sturdy’ planted in autoclaved soil (collected from a field in the Changping district, Beijing). The cysts formed were extracted, and egg suspensions were prepared as described above. Isolated eggs were treated with 0.1% sodium hypochlorite for 2–3 min and rinsed with sterilized water 3 times. Each soil sample was gently but thoroughly mixed with eggs (1500 eggs/100 g of dry soil), and 500 g of treated soil with a moisture content of approximately 10% (v/w) was placed in each PVC pot (9 cm diameter). Two seeds of the variety Sturdy were sown in each pot, which was covered with a polyethylene bag to retain moisture during seed germination. The seedlings were thinned to a single plant after 1 week. The growth chamber was set at 23–25 °C with a 16-h light/8-h dark cycle, and the plants were watered every 2 days. Three replicates (completely randomized) were used for each treatment. Soybean roots harvested 56 days after seeding were stained with fuchsin to measure the degree of penetration by J2s. The density of eggs (the number of eggs/100 g of dry soil) in each pot was measured after plant harvesting, and ANOVA and the LSD test (*p* < 0.05) were used to evaluate significant differences among the treatments.

### Evaluation of rhizosphere and cyst microbiota for their ability to suppress SCN

To determine whether the microbiota associated with cysts and the soybean rhizosphere in S soils contribute to SCN suppressiveness, the preparations noted below were mixed with 450 g of C soil. The following treatments were prepared to evaluate the involvement of cyst-associated microbiota: (i) native SCN cysts directly extracted from 50 g of field S soils, (ii) newly formed cysts in S soils after 56 days of soybean growth following the inoculation of 1500 eggs per 100 g of dry soil, and (iii) cyst suspensions prepared by grinding the cysts from (ii). To evaluate the involvement of soybean rhizosphere microbiota, we performed the following treatments: (i) soybean seedlings grown in the S soil for 2 weeks were transplanted to the C soil, (ii) rhizosphere soils collected from soybean roots prepared as described in (i) as previously described [25], and (iii) soybean rhizosphere and root microbiota suspension prepared by grinding the roots attached with soil particles from (i) in pestle and mortar in the presence of distilled water. C soils were inoculated with 1500 eggs/100 g of dry soil and thoroughly mixed before sowing soybean seeds. Two seeds were sown in each PVC pot (9 cm diameter) filled with 500 g of treated soil, with an initial moisture content of approximately 10% (v/w). Plants were grown in a growth chamber as described above.

### Egg hatching assay

Cysts extracted from SCN-infested field soils and newly-formed cysts extracted from autoclaved soil (collected from a field in the Changping district in Beijing) after growing soybean plants under greenhouse condition (as shown above) were used. Egg hatching rates from the cysts extracted from field soils and the newly formed cysts in the S, C and CS soils or autoclaved soil (control) after 56 days of soybean growth were measured by placing 100 cysts on a micro sieve in 0.05% w/v ZnCl_2_ solution (used to stimulate egg hatching) for 14 days. The total number of juveniles hatched from each cyst sample was counted using an inverted microscope (Olympus CK40). Six-well tissue culture plates were employed for counting. Cysts prepared using autoclaved soil were used to measure egg hatching rates in S and C soil extracts prepared by shaking 100 g of soil in 250 ml distilled water in a flask for 48 h followed by passing the supernatant through 0.22 µm aperture sieve to eliminate microorganisms.

### Cyst bacterial community profiling via amplicon sequencing

To analyse the diversity and abundance of bacteria associated with the cysts formed in the S, C, CS, S80 and SF soils, 30 cysts isolated from each soil were crushed using a glass grinder to release the eggs. DNA was extracted from these samples using the FastDNA Spin Kit for Soil (MP Biomedicals) and the FastPrep instrument (2 min at the speed setting of 6.0). After adjusting the concentration to 20 ng/µL, the V4 region of the 16S rRNA gene was amplified in triplicate reactions using the specific bar-coded primer pair 515F (5′-GTGCCAGCMGCCGCGGTAA-3′) and 806R (5′-GGACTACHVGGGTWTCTAAT-3′) in a Veriti thermal cycler (Applied Biosystems). The PCR condition was initial denaturation at 94 °C for 5 min, followed by 35 cycles of 94 °C for 50 s, 54 °C for 30 s, 72 °C for 40 s, and the final extension at 72 °C for 10 min. Negative control reactions with no DNA were included. The resulting PCR products were separated via agarose gel electrophoresis to purify the amplicons using a DNA Gel Extraction kit (Takara) and pooled to ensure the same concentrations before sequencing. Paired-end sequencing was performed using the Illumina MiSeq sequencer at Allwegene Biotechnology Co., Ltd. (Beijing, China).

### Bioinformatic analyses of amplicon sequencing

Sequences were quality-trimmed using Trimmomatic v0.36 and assigned to individual samples based on their barcodes using QIIME [48]. De novo and reference-based chimaera were checked, and those characterized as chimeric were removed. Sequence reads were binned into operational taxonomic units (OTUs) at the ≥ 97% sequence similarity level using an open-reference OTU picking protocol in the UPARSE pipeline [49], and the most abundant sequences from these OTUs were taken as representative sequences. Taxonomic assignment of these OTUs was performed using the Basic Local Alignment Search Tool (BLAST) against a subset of the Silva database [50].

We calculated the alpha diversity including Shannon’s diversity and the Simpson index on the OTU table using the R package vegan. Analysis of variance and LSD test were used to evaluate the results and compare the treatment mean values. Treatments were considered significant when *p* < 0.05. Venn diagrams were created to identify unique and shared OTUs between different treatments using the R package VennDiagram. The linear regression analysis relating SCN egg densities to the bacterial diversity and specific taxa was conducted using the R package ggplot. Beta diversity calculations were performed on non-rarefied OTU counts. The Bray–Curtis dissimilarity matrix for cluster analysis was calculated using the function vegdist in the vegan R package on ‰ OTU relative abundances log_2_ transformed. The OTUs with a relative abundance (RA) above 1‰ in at least one sample were included in the analysis. The average RA percentage of abundant phyla and families in the rhizosphere and cysts sampled after five soil treatments was calculated based on classified OTU reads and subsequently plotted using the package ggplot2. Analysis of variance and LSD test were used to evaluate the data and compare the treatment mean values. The OTUs enriched in the cysts formed in the S (both Baicheng and Flaerji) and CS soils compared to the cysts formed in the C soils were identified and visualized in ternary plots, in which linear statistics were employed on RA values (log2, > 1‰ threshold) using the Limma R package. Differentially abundant OTUs between groups were identified using a moderate *t*-test, and the obtained *P*-values were adjusted using the Benjamini–Hochberg correction method. Heatmaps were constructed to visualize enriched OTUs using the function heatmap.2 in the gplots.

### Isolation of candidate bacteria, identification, and phylogenetic analysis

100 cysts isolated from each S soil sample were crushed using a glass grinder and resuspended in 2 ml sterile phosphate buffer. After serially diluting the suspension, 100 µl aliquots of 10^−4^ to 10^−7^ dilutions were spread on R2A agar plates using glass beads. After incubating the plates at 25 °C for 2–5 days, distinct bacterial colonies based on morphology, colour and shape were picked and streaked on new R2A agar plates for purification and identified based on their 16S rRNA gene sequence. Cultures of the purified strains in 25% glycerol were stored at − 80 °C. A single colony was picked for colony PCR using 16S rRNA primers. Sequences of each PCR product were quality checked, trimmed and used to search the NCBI nucleotide database. After aligning the 16S rRNA gene sequences using CLUSTAL_X [51], a neighbour-joining phylogenetic tree with the Kimura 2-parameter model was constructed using MEGA 5 with 1000 bootstraps.

### Anti-SCN activity of *Chitinophaga* and *Dyadobacter* isolates

*Chitinophaga* and *Dyadobacter* isolates cultured on R2A plates at 25 °C were used to prepare cell suspensions in 10 mM MgCl_2_ (1 × 10^7^ CFU ml^−1^). Juvenile mortality caused by individual isolates was measured by adding 1 ml cell suspension and 100 J2s to each well of a 12-well tissue culture plate (Nest Biotechnology Co. Ltd), incubating the plate at 25 °C, and counting dead J2s every day for up to 7 days.

To determine whether these strains affected egg hatching, we mixed 20 g of autoclaved C soil with each isolate (1 × 10^7^ CFU/g of soil) and 100 cysts (freshly produced using plants grown in autoclaved soil) and incubated for 7 days. Autoclaved soils mixed with cysts but no bacterial cells served as controls. Cysts in the treated soils were recovered as described above and placed on a micro sieve in ZnCl_2_ solution. Egg hatching was measured in a 12-well tissue culture plate using an inverted microscope. SCN eggs and juveniles were photographed using a Nikon ECLIPSE 80i compound microscope equipped with a Canon EOS 600D digital camera, and the resulting images were recorded using the ImageAnalysisSystem11 image processing software.

### Colonization of the SCN cyst and female by *Chitinophaga* and *Dyadobacter*

Cells of each *Chitinophaga* and *Dyadobacter* isolate (1 × 10^7^ CFU/g of dry soil) and SCN eggs (1500 eggs/100 g of dry soil) were gently but thoroughly mixed with autoclaved C soil. After growing plants using treated soils for 56 days, as described above, female and cyst samples were collected from soybean roots and crushed using a glass grinder containing 1 ml of 100 mM sodium phosphate buffer at pH 7. The resulting suspensions were transferred to 1.5 ml tubes. A series of tenfold dilutions made using 100 mM sodium phosphate buffer (pH 7), with thorough vortexing before each dilution, were plated onto R2A and incubated at 25 °C to determine CFU.

### Measurement of chitinase activity in *Chitinophaga*

Chitinase activity of *Chitinophaga* isolates CN7 and C54 was measured using a kit based on the dinitrosalicylic acid (DNS) method following the manufacturer’s instruction (Nanjing Jiancheng Bioengineering Institute, Nanjing, China). After incubation, the bacteria cell-free culture broth was prepared by centrifugation at 10,000 rpm for 15 min, and the reaction was stopped by placing the reaction tube at 100 °C for 5 min and centrifugated at 8000 rpm for 10 min at 4 °C. After diluting the supernatant tenfold, 700 μl of the diluted supernatant was mixed with 0.5 μl DNS and heated at 100 °C for 10 min. Absorbance at 540 nm was measured using a Fluoroskan Ascent FL microplate reader (Thermo Fisher, Waltham, MA, USA). One unit (U) of enzyme activity, defined as the amount of enzyme needed to release 1 mg of N-acetyl-glucosamine per hour and expressed as U ml^−1^.

### Whole genome sequencing of *Chitinophaga* isolates CN7 and C54

Genomic DNA from the *Chitinophaga* isolates CN7 and C54 was extracted using a SDS-based method. The extracted DNA was visualized using agarose gel electrophoresis and quantified using a Qubit 2.0 Fluorometer. Sequencing libraries were generated using the NEBNext Ultra DNA Library Prep Kit for Illumina following the manufacturer’s recommendations, and index codes were added to attribute sequences to each sample. Whole genomes of CN7 and C54 were sequenced using the PacBio Sequel platform and Illumina HiSeq/ NovaSeq PE150 by Allwegene Biotechnology Co., Ltd. (Beijing, China). Genome assembly was performed using the SMRT Link v5.0.1. Hereafter, the prediction of the components of coding genes and functional annotation using various databases such as NR, CAZy and COG were performed.

### Real-time qRT-PCR analysis of transcripts from *Chitinophaga* GH18 genes

*Chitinophaga* isolate CN7 cells (10^9^ CFU/ml) were added to each well containing 100 SCN eggs in 12-well tissue culture plates and incubated for 0, 24 and 48 h. Bacterial cells cultured in the absence of SCN eggs were used as controls. Bacterial RNAs were extracted using the Zhuangming U-Fast bacterial RNA extraction kit following the manufacturer’s instruction. After measuring the RNA concentration, 1 μg of RNAs was converted to complementary DNAs (cDNA) using a Tiangen reagent kit. SYBR Green PCR master mix (Takara, Mountain View, CA, USA) was used for quantitative RT-PCR using ABI Prism 7500 (Life Technologies, Carlsbad, CA, USA) with the programs recommended by the manufacturer. The primers used for amplifying three glycosyl hydrolase 18 (GH18) genes of *Chitinophaga*, *chpbs_4014*, *chpbs_4904*, and *chpbs_5684,* are listed in Table S2. The *glyA* gene was used as an internal control. The threshold value (CT) was used to quantify relative gene expression levels using the comparative 2^−ΔΔCT^ method.

### Cloning, expression and partial purification of *Chitinophaga* GH18 genes

Three GH18 genes of *Chitinophaga*, including *chpbs_4014*, *chpbs_4904*, and *chpbs_5684*, were cloned into the MCS of pET28a using the ClonExpress II one-step cloning kit (Vazyme) to construct protein expression vectors (the primers used are listed in Table S3), and the vectors were transformed into *E. coli* BL21 for protein expression. Transformants were inoculated into LB containing 40 μg/ml kanamycin, incubated at 37 °C until the OD_600_ value reached 0.8, and then induced with IPTG (final concentration of 0.1 mM) for 20 h at 16 °C. The cultures were collected by centrifugation, resuspended in the buffer [25 mM Tris–HCl, 200 mM NaCl; pH = 7.8], and disrupted by ultrasonication. After centrifugation at 12,000 rpm for 30 min at 4 °C, the supernatants were transferred to a Ni^2+^-nitrilotriacetic acid (NTA) column and incubated at 4 °C for 2 h. After pre-experiment, we used 50 mM imidazole buffer (25 mM Tris–HCl, 200 mM NaCl, 50 mM imidazole; pH = 7.8) to elute non-target proteins, and 150 mM imidazole buffer (25 mM Tris–HCl, 200 mM NaCl, 150 mM imidazole; pH = 7.8) was used to elute proteins with His tag. The collected proteins were assayed for protein concentration using an ultra-micro spectrophotometer and detected by SDS-PAGE.

### Real-time qRT-PCR analysis of transcripts from soybean defence-related genes

To determine whether *Dyadobacter* D18C cells attached to surface coat of J2s induce host defence responses during root invasion, we quantified transcripts from selected defence-related genes in roots of soybean plants grown in twice autoclaved soils after treating with ~ 2000 J2s, J2s attached with D18C (after co-incubating freshly prepared J2s with ~ 2000 D18C cells overnight, the J2s were collected using 25 µm sieve), D18C cells (~ 1 × 10^7^ cells/g of soil), and 5 ml of 50 μM PBS buffer, pH 7.0 (control). Each treatment in 5 ml of 50 μM PBS buffer was applied to the soil near the roots of 7-day-old plant (6 biological replicates). The treated roots were gently removed from the soil 7 days later, thoroughly rinsed with tap water to remove attached soil, air dried on paper tissues, weighed, and divided into two portions. One portion (approximately 0.25 g) was wrapped in aluminium foil, treated with liquid nitrogen, and stored at − 80 °C until RNA extraction. The remaining roots were stained with fuchsin to assess the degree of penetration by J2s. Total RNAs were isolated using the RNAprep Pure Plant Kit (Tiangen, Beijing, China), and 1 μg of RNAs was converted to cDNA using the Superscript III Reverse Transcription Kit (Invitrogen). SYBR Green PCR master mix (Takara) was used for quantitative RT-PCR using ABI Prism 7500 with the programs recommended by the manufacturer. The primers used are listed in Table S4. The actin gene was used as an internal control. Threshold value (CT) was employed to quantify relative gene expression levels using a comparative 2^−ΔΔCT^ method.

### Effect of *Chitinophaga* and *Dyadobacter* on egg density and hatching

The C soil was inoculated with *Chitinophaga* CN7 and C54 and *Dyadobacter* D18C individually and in combination (CN7/C54, CN7/D18C, C54/D18C, and CN7/C54/D18C) to determine whether they suppress SCN. For all inoculations, regardless of the number of strains used, the cell density was 1 × 10^7^ CFU/g of soil. After thoroughly mixing the soil inoculated with bacterial cells, 1500 eggs (per 100 g of dry soil) were gently but thoroughly mixed with the inoculated soil. Three PVC pots filled with each treated soil were used to grow soybean plants as noted above. The plants were harvested 56 days after seeding. The egg density was measured, and ANOVA and LSD test (*p* < 0.05) were used to evaluate significant differences among the treatments. This experiment was repeated twice. We grew a new soybean plant in the pots used for evaluating *Chitinophaga* isolates without adding a new batch of bacterial cells to determine if they could continuously suppress egg hatching. After another 56 days of soybean growth, we measured the egg density and hatching, and ANOVA and LSD test (*p* < 0.05) were used to evaluate significant differences among the treatments.

### Supplementary Information

Supplementary Material 1: Table S1. Physical and chemical properties of SCN suppressive and conducive field soils used in this study. Table S2. Primers used to quantify the transcript levels of GH18 genes of Chitinophaga isolate CN7 by qRT-PCR. Table S3. Primers used to amplify three GH18 genes of Chitinophaga isolate CN7 for protein production in Escherichia coli. Table S4. Primers used to quantify the transcript levels of selected defense-related genes in soybean via qRT-PCR. Fig. S1. SCN egg densities and soybean growth in the suppressive (S) and conducive (C) soils collected from soybean fields in Baicheng and Flaerji. A. Egg densities in the S and C soils. B. Soybean shoot weight after 56 days of growth using the S and C soils in a growth chamber. C. Cyst densities and soybean shoot weight after 56 days of growth in the following soils inoculated with 1,500 eggs per 100g of dried soil: S and C soils, transferred soil (CS; created by mixing 10% S soil with 90% C soil), heat (80°C)-treated S soil (S80), and S soil fumigated with formalin (SF). Different letters above the bars denote statistically significant differences according to LSD test (*p* < 0.05). Fig. S2. SCN suppression by different field soils and soil treatments. Greenhouse trials were performed to determine how various soils and soil treatments affect SCN egg densities and whether bacteria and fungi in the rhizosphere and SCN cyst contribute to suppressing SCN. The suppressive (S) and conducive (C) soils collected from Baicheng (A) and Fulaerji (B) were used. The egg densities were measured after 56 days growth of soybean with inoculation of 1,500 eggs/100g dried soil. The classical trial for suppressive soil included the treatments: suppressive (S) soil, conducive (C) soil, transferred soil (CS; created by mixing 10% S soil with 90% C soil), heat (80°C)-treated S soil (S80), and S soil fumigated with formalin (SF). To illustrate contributions of cysts (Cy) and soybean rhizosphere (Rh) microbiota in SCN suppressiveness, microbial suspensions were prepared from the cysts and rhizosphere in the S soil equal to 10% amount S soil in CS treatment and then mixed with 90% C soil. The rhizosphere microbial suspensions were prepared by collected rhizosphere (including roots) soil from soybean seedlings growing in S soil equal to 10% S soil in CS treatment for two weeks and ground in distilled water (CS-Rh). Cysts microbial suspensions were created by extracting cysts from native S soil equal to amount of 10% S soil in the CS treatment and ground in distilled water (CS-Cy). The microbial suspensions were also treated with antibiotics to kill fungi (pimafucin=200 μg/mL) and remain bacteria (CS-Rh-bacteria or CS-Cy-bacteria) or to kill bacteria (penicillin=100 U/mL+streptomycin=100 μg/mL) and remain fungi (CS-Rh-fungi or CS-Cy-fungi) or to kill all microbiota by 3 antibiotics (CS-Rh-antibiotics or CS-Cy-antibiotics). Different letters above the bars denote statistically significant differences according to LSD test (*p* < 0.05). Fig. S3. Within-sample diversity (α-diversity) of bacterial community inhabiting the cysts isolated from five soil treatments. A. Bacterial community cluster analysis based on Bray–Curtis dissimilarity showed clear separation between SCN cyst in S and CS soil treatments than C, S80 and SF (left in Baicheng and Flaerji). The horizontal bar within each box indicates median value. The tops and bottoms of boxes represent 75th and 25th quartiles, respectively. Linear regression relationship between the cyst bacteria diversity and egg densities. Regression line is in brown, and the shaded region represents the confidence interval (geom_smooth function, method = lm). B. Cluster analysis based on Bray–Curtis dissimilarity indicates clear separation between bacterial communities inhabiting the SCN cyst in S and CS soil treatments than C, S80, and SF. Bacterial OTUs with RA > 1‰ in at least one sample were included in the analysis. Fig. S4. Averaged relative abundance (RA) and linear regression relationship between the most dominant bacterial phyla and the egg density. Data from analysing the S and C field soils collected in (A) Baicheng and (B) Fulaeji were used for this analysis. The brown line and the shaded region on the right side indicate the regression line and the confidence interval (geom_smooth function, method = lm), respectively. Only the taxa with RA of > 1% in at least one sample were included in the analysis. Fig. S5. Average relative abundance (RA) of the most dominant bacterial families associated with the cysts formed in different soils. Chitinophagaceae was more abundant in the cysts formed in the S and CS soils than those formed in the C soils collected from two provinces (A=Beicheng, B=Fulaerji). Only taxa with RA of > 1% in at least one sample were included. Fig. S6. Ternary plots and heatmaps demonstrating the OTUs significantly enriched in heat (S80)- and formalin (SF)-treated soils compared to S across two district geographical locations (Baicheng-BC, A and B; Fulaerji-FL, C and D). Dark golden circles mark cyst OTUs significantly enriched in S80 than S (S80 > S OTUs). Pink circles mark cyst OTUs significantly enriched in SF than S (SF > S OTUs). Green circles mark cyst OTUs simultaneously enriched in S80 and SF compared to S (S80+SF > S OTUs). Each circle represents one OTU. The size of each circle represents its relative abundance. The position of each circle is determined by the contribution of the indicated compartments to the total relative abundance. Only taxa with RA > 1‰ in at least one sample were included in the analysis. Fig. S7. Detection and quantification of Chitinophaga in the suppressive soil and cysts by qPCR. A. The gel image shows specific amplification of the targeted Chitinophaga DNA region using the primer pair hu3135254F (5′-CATTGAGAGGCATCTTTTG-3′) and hu1185451R (5′-CGGTGCTTATTCATCTGGTA-3′). M indicates DNA size markers (2000 bp). Lanes 1-6 show PCR amplification products in the absence of Chitinophaga genomic DNA. Lanes 7-12 show PCR amplification products in the presence of Chitinophaga genomic DNA. B. Estimation of the percentage of Chitinophaga among the bacterial cells in the suppressive soil and those associated with cysts. The primers 27F and 1492R, designed to amply the bacterial 16S rRNA genes, were used to quantify the total bacterial community size in each sample. (C) Chitinophaga RA % in rhizosphere, root and cyst of suppressive soils challenged with nematodes in a previous study by Hussain et al. [1]. Fig. S8. The circos plot displaying the genomic features of chromosomal level Chitinophaga isolate CN7 genome. COG functional gene distribution. The legend is shown on the right side. Fig. S9. The circos plot displaying the genomic features of chromosomal level Chitinophaga isolates C54 genome. COG functional gene distribution. The legend is shown on the right side. Fig. S10. Relative expression of chitinases encoded by three glycosyl hydrolase 18 (GH18) genes of Chitinophaga isolate CN7 challenged by SCN eggs and their heterogeneous expression using Escherichia coli BL21 and their purification. The PCR primers used for expression vector construction are listed in Table S3. A. Expression of GH18 genes after 0, 24 and 48 h by inoculation of 1 ml CN7 cell suspension (10^9^ cfu/mL) to 100 SCN eggs. B. SDS-PAGE of cell lysates before and after IPTG induction. The bands corresponding to target proteins were marked in black boxes. Proteins in the whole cell lysate (A) and the supernatant (S) and precipitate (R) of the lysate after ultra-sonication and centrifugation were analyzed. C. SDS-PAGE of purified proteins. The target protein bands are marked in black box. M=molecular weight (kDa) markers; T=the cell lysate before passing through Ni-NTA; 1-2=elutants with 50 mM imidazole buffer; 3-7=elutants with 150 mM imidazole buffer; 8=elutant with 500 mM imidazole buffer.

## Data Availability

All raw data associated with this study are available in the supplementary materials. Bacteria isolates 16S rRNA gene sequences have been deposited to the NCBI database under accession no. OQ439404-OQ439409. All raw data from 16S rRNA gene amplicon sequencing were deposited in the NCBI SRA with accession no. PRJNA911970 (https://dataview.ncbi.nlm.nih.gov/object/PRJNA911970?reviewer=sa0mjhmrgsjg876unddr0rusjc).
